# Preprocedural ultrasound versus landmark techniques for spinal anesthesia performed by novice residents in elderly: a randomized controlled trial

**DOI:** 10.1186/s12871-019-0882-8

**Published:** 2019-11-11

**Authors:** Marwan S. Rizk, Carine A. Zeeni, Joanna N. Bouez, Nathalie J. Bteich, Samia K. Sayyid, Waseem S. Alfahel, Sahar M. Siddik-Sayyid

**Affiliations:** 1 Department of Anesthesiology, American University of Beirut Medical Center, P.O. Box 11-0236, Beirut, Lebanon; 2 Division of Musculoskeletal Imaging, Emory University Hospital, Atlanta, USA

**Keywords:** Spinal anesthesia, Ultrasound imaging, Geriatric

## Abstract

**Background:**

Spinal anesthesia using the midline approach might be technically difficult in geriatric population. We hypothesized that pre-procedural ultrasound (US)-guided paramedian technique and pre-procedural US-guided midline technique would result in a different spinal anesthesia success rate at first attempt when compared with the conventional landmark-guided midline technique in elderly patients.

**Methods:**

In this prospective, randomized, controlled study*,* one hundred-eighty consenting patients scheduled for elective surgery were randomized into the conventional surface landmark-guided midline technique (group LM), the pre-procedural US-guided paramedian technique (group UP), or the pre-procedural US-guided midline technique (group UM) with 60 patients in each group. All spinal anesthesia were performed by a novice resident.

**Results:**

The successful dural puncture rate on first attempt (primary outcome) was higher in groups LM and UM (77 and 73% respectively) than in group UP (42%; *P* < 0.001). The median number of attempts was lower in groups LM and UM (1 [1] and 1 [1–1.75] respectively) than in group UP (2 [1, 2]; *P* < 0.001). The median number of passes was lower in groups LM and UM (2 [0.25–3] and 2 [0–4]; respectively) than in group UP (4 [2–7.75]; *P* < 0.001). The time taken to perform the spinal anesthesia was not different between groups LM and UM (87.24 ± 79.51 s and 116.32 ± 98.12 s, respectively) but shorter than in group UP (154.58 ± 91.51 s; *P* < 0.001).

**Conclusions:**

A pre-procedural US scan did not improve the ease of midline and paramedian spinal anesthesia as compared to the conventional landmark midline technique when performed by junior residents in elderly population.

**Trial registration:**

Retrospectively registered at Clinicaltrials.gov, registration number NCT02658058, date of registration: January 18, 2016.

## Background

Spinal anesthesia is traditionally performed using the palpation of bony landmarks to identify the level and point of the needle insertion, together with haptic feedback during needle insertion. Ultrasound (US) imaging has become an increasingly popular tool among anesthesiologists to guide neuraxial blockade. Studies have shown that pre-procedural US facilitates the performance of spinal anesthesia in patients in whom technical difficulties are expected [1, 2] and is not of significant benefit over the traditional landmark technique when it is performed for patients without lumbar spine abnormalities [3–5].

According to a practice survey amongst UK anesthesiologists, the conventional midline approach is the most commonly used technique for spinal anesthesia [6]. However, the paramedian palpation approach has shown to be useful in geriatric population, since it is less influenced by spinal osteoarthritic changes, and is associated with a higher success rate than palpation midline approach in the elderly (85% vs. 45%) [7].

Most of the studies on pre-procedural US-guided neuraxial techniques are limited to a midline approach using a transverse median (TM) view. The parasagittal oblique (PSO) view allows for a wider ultrasound window of the epidural space, providing an enhanced visibility of the neuraxis and surrounding structures compared to the TM view [8]. However, it is still not evident whether these superior PSO views lead to an easier paramedian needle insertion. In the literature, there are no studies directly comparing the US-guided paramedian approach using the PSO view, the US-guided midline approach using the TM view, and the conventional landmark midline approach to perform spinal anesthesia by novice residents in elderly patients.

In this prospective, randomized, controlled study, we hypothesized that both midline and paramedian pre-procedural US-guided spinal anesthesia would result in different success rates at first attempt when compared with the conventional landmark-guided midline technique in elderly patients. All procedures were executed by first year clinical anesthesia residents (CA-1) under direct staff anesthesiologist supervision.

## Methods

This study was approved by the American University of Beirut Institutional Review Board and written informed consent was obtained from all patients and from the 14 residents participating in the trial. The study adheres to the CONSORT guidelines and was retrospectively registered at clinicaltrials.gov (NCT02658058, principal investigator: Sahar Siddik-Sayyid, date of registration: January 18, 2016).

Patients scheduled for surgery under spinal anesthesia, were more than 60 years old, with American Society of Anesthesiologists physical status 1 to 4, were considered eligible for enrollment. Patients who were unable to give informed consent, refused spinal anesthesia or had contraindications to spinal anesthesia, including allergy to local anesthetics or a bleeding diathesis were excluded.

After obtaining informed consent, a computer-generated block randomization schedule was used to randomize patients to receive spinal anesthesia into one of three treatment groups: the conventional surface landmark-guided midline technique (group LM), the pre-procedural US-guided paramedian technique (group UP), or the pre-procedural US-guided midline technique (group UM). Group allocation was concealed from study investigators until the procedure time. Due to the nature of the study, blinding of the residents performing the procedure and observer collecting data was not possible. Only patients were blinded to the study group.

Baseline patients characteristics recorded were: age, gender, body mass index, and presence of any spinal abnormalities (including significant scoliosis on physical exam and previous spine operations with instrumentation). Upon arrival to the operating room, standard monitoring (three-lead electrocardiogram, noninvasive blood pressure, and pulse oximeter) and intravenous access were established. The operator performing the procedure was a CA-1 under direct supervision of one attending anesthesiologist (MR) with fifteen years of clinical experience. All US imaging of the lumbar spine were performed by the same attending anesthesiologist trained and experienced in US-assisted neuraxial block. The Sonosite (TM, Bothell, WA 98021 USA) with a low frequency (2 to 5 MHz) curvilinear probe was used for this study. The pre-procedural spinal US was performed in a nonsterile manner. Thereafter, all spinal procedures were carried out with the patient in the sitting position and under sterile technique. All patients were requested to maintain a lumbar flexion posture. The lumbar interspaces selected were presumably between L2 and L5.

Each resident was randomly allocated procedures in subject allocation blocks of six. Each subject allocation block contained randomly two landmark-guided midline techniques, two pre-procedural US-guided paramedian techniques, and two pre-procedural US-guided midline techniques. Each resident did six spinal blocks in a row and had to complete two to three subject allocation blocks. Residents chosen were novices who had performed less than five spinal attempts since the beginning of their residency. They were instructed about the three spinal techniques by watching 3 cases of each before the beginning of the study, in addition to the standardized teaching about spinal anesthesia that included teaching videos and reading material.

### Study interventions

In group LM, spinal anesthesia was performed using the conventional surface anatomic landmark-guided technique and a midline approach. The resident palpated first the surface anatomic landmarks (iliac crests, lumbar spinous processes and interspinous spaces) with landmark identification confirmed by the attending anesthesiologist. The quality of surface landmarks was graded by the attending anesthesiologist according to the overall ease of palpation on a 4-point scale: easy, moderate, difficult or impossible. The lumbar interspace that appeared widest was chosen for the first attempt, and the site of needle insertion was marked on the patient’s skin.

In both US groups, the resident palpated the surface anatomical landmarks, and the quality was graded as described above. Then the investigator (MR) performed the pre-procedural US examination, demonstrating explicitly the lumbar spine view for the resident (who was present at all times during US visualization). The quality of the scan at each level was recorded and the level at which it was optimal was chosen as the interspace for the first attempt. Also, the PSO and TM views were graded as good (both the ligamentum flavum-dura mater complex (LFD) and posterior longitudinal ligament (PLL) visible), intermediate (either LFD or PLL visible), or poor (both LFD and PLL not visible).

In group UP, the probe was oriented longitudinally to obtain a parasagittal oblique view of the lumbosacral spine, in which the L2–L3 to L4–L5 interlaminar spaces were identified by counting upward from the sacrum. The locations of the interlaminar spaces were identified by visualizing the LFD and the PLL. The angulation at which LFD and PLL were best visualized was considered the optimal angle for needle insertion, and was clearly communicated to the resident, in addition to the distance from skin to dura. The interlaminar space was then centered on the US screen and a skin mark was made on the patient’s back at the intersection point of 2 lines joining the midpoints of long and short borders of the probe.

In group UM, the transducer was applied in the parasagittal plane, and after identification of the intervertebral levels as described above, the probe was rotated 90 degrees to obtain the TM view. Similarly, the angle at which the LFD and PLL were best visualized was noted. The resident was also informed about the direction of the probe and depth to the dura. A skin mark was placed on the patient’s back at the intersection point of 2 lines joining the midpoints of long and short borders of the probe.

For all three groups, if the first attempt was unsuccessful, further attempts could be made at the same interspace. No more than 3 attempts were permitted to the residents, after which the attending anesthesiologist was given the option to use an alternative technique and/or another interspace. All residents used a 25 G Whitacre 90-mm, pencil-point spinal needle through a 20 gauge introducer, and patients received heavy bupivacaine 0.5% (12–15 mg).

### Data collection

The primary outcome measure was the rate of successful dural puncture on the first needle insertion attempt. Any additional needle attempt is defined as a complete withdrawal of the introducer needle from the skin and subsequent reinsertion. This differs from a needle redirection which is defined as an incomplete withdrawal of the needle from the patient’s skin and change in its insertion path.

The secondary outcomes included the following: number of needle insertion attempts required for successful dural puncture, number of needle passes (insertion + redirection attempts required for successful dural puncture), time taken to perform the spinal anesthesia (defined as the time from the first insertion of the introducer needle till withdrawal of the spinal needle after intrathecal injection of the anesthetic solution), patient satisfaction (rated immediately after spinal block completion as very good, good, or satisfactory), peri-procedural pain score (rated by patients immediately after spinal block completion on a scale from 0 to 10), success of spinal anesthesia (defined as a sensory block level above T_10_ within 30 min of administration of the local anesthetic), requirement for verbal assistance by the attending anesthesiologist while the resident is doing the spinal block, and complications such as bloody tap or paresthesia.

All data were measured and recorded by one of the research team members who was not involved in the case’s anesthetic management.

### Statistics

Sample size calculation was based on the aim to improve successful dural puncture on the first needle insertion attempt (the primary outcome) from 60% with the landmark-guided technique to 84% with the pre-procedural US-guided techniques, as per a recent study in the elderly population [9]. The used method was JavaStat -- Binomial Proportion Differences (https://statpages.info/proppowr.html). We concluded that 54 patients would be required in each group to achieve a power of 0.8 and a type 1 error rate of less than 0.05. The sample size was increased to 60 per group to compensate for potential subject loss that may occur during the course of the study (180 patients in total).

The primary outcome (successful dural puncture on first attempt) was expressed as numbers and percentages and was analyzed using Chi square or Fisher’s exact test as appropriate. For secondary outcomes, categorical data (ease of landmark palpation, grading by US, successful dural puncture, successful dural puncture on first pass, patient satisfaction, verbal attending assistance, and complications) were reported as numbers and percentages and were analyzed using Chi square or Fisher’s exact test as appropriate. Non parametric data (number of attempts, number of passes, and pain scores) were reported as medians and interquartile ranges and were analyzed using Mann-Whitney U-test. Continuous data (time taken to perform spinal anesthesia) were reported as means ± standard deviations and were analyzed using ANOVA test using Tukey. *P* < 0.05 was considered significant. We used SPSS version 23 (SPSS Inc., Chicago, IL) for our statistical analysis.

## Results

**Fig. 1:**
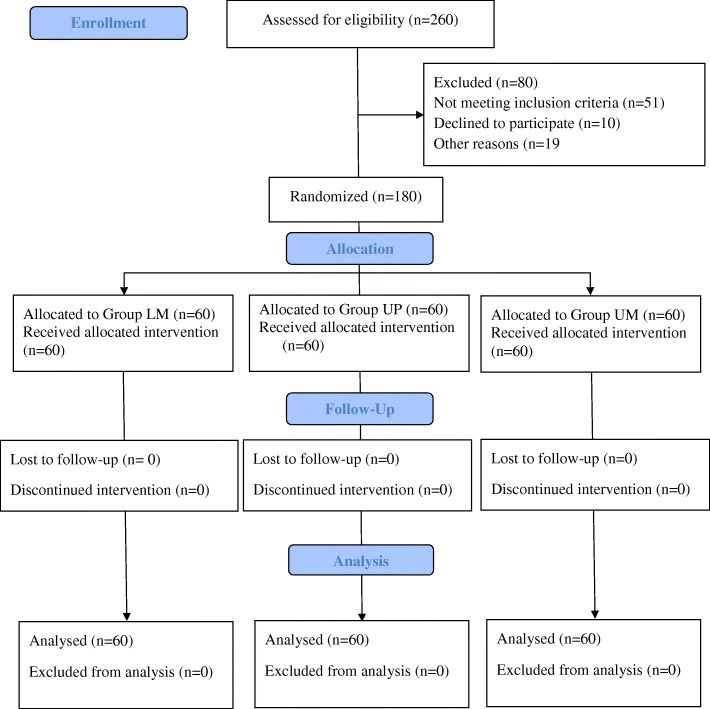
Consort flow diagram

Twelve of the 14 residents did two subject allocation blocks (4 procedures of each group) and the remaining two did 3 subject allocation blocks (6 procedures of each group). We randomized 209 patients of whom twenty-nine did not receive the allocated intervention for the following reasons: the surgical procedure was canceled by the surgical team (4 patients), the attending anesthesiologist with the expertise in the US-guided technique was unavailable (11 patients), it was deemed that there was insufficient time to perform study assessments (3 patients), or there was a change in the anesthesia type (11 patients). The final number of patients included was 60 patients for each group (Fig. 1). No patients were lost to follow-up. The three groups were similar regarding baseline demographics and type of surgery (Table 1). None of our patients had scoliosis or previous spine operations.

**Table 1:** Baseline characteristics

	Group LM (*n* = 60)	Group UM (*n* = 60)	Group UP (*n* = 60)
Age (y)	73.51 ± 7.99	72.37 ± 7.83	71.42 ± 7.54
Weight (kg)	77.72 ± 11.48	81.73 ± 17.09	78.44 ± 15.13
Height (cm)	166.13 ± 8.86	167.83 ± 7.12	167.48 ± 8.19
BMI (kg/m^2^)	28.21 ± 4.30	28.98 ± 6.01	28.21 ± 4.67
Gender			
M	47 (78)	50 (83)	49 (82)
F	13 (22)	10 (17)	11 (18)
Type of surgery			
Urology	47 (78)	47 (78)	51 (85)
Orthopedics	6 (10)	7 (12)	8 (13)
General surgery	7 (12)	6 (10)	1 (2)

Values are mean ± SD or numbers (%)

*Group LM* landmark-guided midline technique, group, *UP* Ultrasound-guided paramedian technique, group, *UM* Ultrasound -guided midline technique

The successful dural puncture rate on first attempt (primary outcome) was higher in groups LM and UM (77 and 73% respectively) than in group UP (42%; *P* < 0.001). The median number of attempts was lower in groups LM and UM (1 [1] and 1 [1–1.75] respectively) than in group UP (2 [1–2]; *P* < 0.001). Also, the median number of passes was lower in groups LM and UM (2 [0.25–3] and 2 [0–4]; respectively) than in group UP (4 [2–7.75]; *P* < 0.001) (Table 2).

**Table 2:** Clinical outcomes

	Group LM (*n* = 60)	Group UP (*n* = 60)	Group UM (*n* = 60)	*P*
Primary outcome				
Successful dural puncture on first attempt	46 (77) *P < 0.001	25 (42)	44 (73) †*P* < 0.001	< 0.001
Secondary Outcomes				
Successful dural puncture on first pass	15 (33)	5 (20)	19 (43)	0.14
Successfull dural puncture	53 (88) *P = 0.001	38 (63)	48 (80) †*P* = 0.043	< 0.001
Number of attempts	1 (1–1) * *P* = 0.001	2 (1–2)	1 (1–1.75) † *P* = 0.003	0.001
Number of passes	2 (0.25–3) *P < 0.001	4 (2–7.75)	2 (0–4) † *P* < 0.001	< 0.001
Time taken to perform spinal anesthesia (s)	87.24 ± 79.51 *P < 0.001	154.58 ± 91.51	116.32 ± 98.12 † *P* = 0.006	< 0.001
Ease of landmark palpation				0.89
Easy	38 (63)	37 (62)	36 (60)	
Moderate	19 (32)	18 (30)	18 (30)	
Difficult	3 (5)	5 (8)	6 (10)	
Impossible	0 (0)	0 (0)	0 (0)	
Grading by ultrasound				0.50
Good		40 (70)	34 (65)	
Intermediate		16 (28)	18 (35)	
Poor		1 (2)	0 (0)	
Verbal attending assistance	32 (53) *P < 0.001	51 (85)	29 (48) † *P* < 0.001	< 0.001
Number of patients who developed complications	10 (17)	20 (33)	9 (15)	0.026
Number of complications	10	26	9	
Paresthesia	0	3	0	
Blood tap	9	15	8	
Radicular pain	1 * *P* = 0.035	8	1 † *P* = 0.019	
Periprocedural pain score	0 (0–0) * *P* = 0.009	0 (0–1)	0 (0–1) † *P* = 0.042	0.017
Satisfaction				0.002
Very good	51 (85.00)	31 (51.67)	44 (73.33)	
Good	8 (13.33)	27 (45.00)	15 (25.00)	
Satisfactory	1 (1.67) * *P* = 0.0004	2 (3.33)	1 (1.67) † *P* = 0.049	

Values are mean ± SD, numbers (%), or medians and interquartile ranges

*Group LM* landmark-guided midline technique, group, *UP* Ultrasound-guided paramedian technique, group, *UM* Ultrasound -guided midline technique

* Group LM vs Group UP; † Group UM vs Group UP

The ease of landmark palpation was similar between the three groups, and grading by US was similar between the US groups. The time taken to perform the spinal anesthetic was not different between groups LM and UM (87.24 ± 79.51 sand 116.32 ± 98.12 s respectively) but shorter than in group UP (154.58 ± 91.51 s; *P* < 0.001). Less verbal attending assistance was required in groups LM and UM (53 and 48% respectively) compared to group UP (85%; *P* < 0.001) (Table 2).

More complications (paresthesia, blood tap, and radicular pain) occurred in patients in group UP, and patients with complications were followed-up to 24 h as per hospital protocol with no consequences. Also, percentage of patients satisfied during the procedure was less in group UP (Table 2). All spinal anesthetics were successful and patients achieved complete sensory block to the T_7_ dermatome or higher. All failed dural punctures by the residents were achieved successfully by the attending anesthesiologist using the same technique as the resident except for one patient in group UP and three patients in group UM who had the spinal procedure using the LM technique.

## Discussion

Our study showed that both preprocedural US-assisted modalities (midline or paramedian) did not prove more efficacious than the landmark-based midline approach in facilitating spinal anesthesia performed by CA-1 residents in elderly patients. In fact, while the first attempt and overall success rates of dural puncture in the midline US and the conventional landmark groups were not significantly different, they were higher than the rates achieved with preprocedural US-guided paramedian spinal technique. Furthermore, time to perform spinal anesthesia, need for verbal attending assistance, as well as the number of attempts and passes were all significantly less in the midline US and conventional groups compared to the paramedian US group.

Metanalyses suggest that preprocedural US leads to reduction of the risk of failure and a lower number of needle passes compared to conventional palpation approach [10–12]. This is particularly true in patients with whom technical difficulties are expected such as those with high body mass indices, nonpalpable landmarks, or difficult spinal anatomy. Of note, most of these studies are limited to a midline approach using a TM view.

Geriatric population, similar to our study population, also may present with higher likelihood of technical difficulties during spinal anesthesia due to narrowed interspinous spaces and interlaminar spaces as a result of ossification of the interspinous ligaments and hypertrophy of the facet joints respectively [1]. However, our findings did not demonstrate an improved outcome with the US midline vs landmark. This may be explained by the fact that the spinal procedures in our elderly population using the landmark midline technique were not difficult enough and easier than expected, thus limiting the benefit of a preprocedural US. In fact, the first success rate in the LM and UM groups (77 and 73% respectively) were higher than that reported in a previous study conducted by Chin et al. in a nonobstetric patient population with difficult anatomic landmarks (32% in landmark group and 65% in US group), even though operator in all cases in the aforementioned study was a clinical fellow in regional anesthesia or consultant with more than 5 year clinical experience [1].

As for the paramedian approach, it should theoretically be valuable in the elderly since the interlaminar spaces are less affected by aging offering a better view of LFD and PLL compared to midline view [13], and it does not require flexing of the spine (an advantage in elderly patients with fractures). Even with these advantages, a lower first attempt success rate with the paramedian approach was obtained compared to both landmark- and US-guided midline approaches. As a matter of fact, the paramedian approach has been shown to be superior to the midline approach in some previous studies [7, 14, 15] but not in others [16–18]. It must be noted that we found only two studies in the literature that describe the use of paramedian US techniques to facilitate spinal anesthesia with contradictory results [4, 9]. Lim et al. compared an US-assisted paramedian vs a conventional paravertebral approach for orthopedic and other types of surgery and found no intergroup difference in first skin puncture-success rate, number of needle redirections, and complications [4]. In another study, Srinivasan et al. compared an US-assisted paramedian vs a palpation at midline approach for an elderly orthopedic population and showed that US technique required fewer passes and attempts to reach the subarachnoid space [9]. We found a much lower success rate at first attempt in the US paramedian group (42%) compared to those in the Lim et al. and Srinivasan et al. studies (64% vs 84% respectively). This may be explained by that our operators were CA-1 residents as opposed to the other two studies where operators were either trainees at variable level of experience (up to three years of experience) [4] or attending anesthesiologists [9].

In our study, the higher number of attempts in the UP group resulted in more blood taps, paresthesia, and radicular pain than in the other two groups. Also, patients in the UP group had more periprocedural pain and lower satisfaction than the other two groups, probably because the needle has to cross the erector spinae muscle before reaching the dura. These findings can be due to the fact that the paramedian approach to the neuraxis is intrinsically more difficult to perform and is utilized by few anesthesiologists.

Studies with junior residents acting as operators during preprocedural US scanning of the spine are present in the obstetric literature with mixed results. All investigators used the midline approach prior to the spinal block. While Sahin et al. found a high level of success in the prepuncture US-determined insertion point by anesthesia residents [2], Turkstra et al. reported similar results to our findings with no observed benefit to preprocedural US examination for junior residents performing spinal anesthesia [5].

Limitations in this study include lack of blinding of the residents and observer collecting data causing a potential bias. Second, the design of the study did not completely eliminate both patient and operator bias. However, we assumed that computer-generated randomization would equally distribute patients of different levels of spinal anesthesia difficulty and thus would decrease patient bias. Anyhow*,* results showed that none of our patients had scoliosis or previous spine operations, both of which are common features that would further increase the difficulty of spinal anesthesia. In addition, the ease of landmark palpation was not different among the three groups. Although operator bias cannot be ruled out completely, we believe that our operator group is a homogenous one consisting of junior residents with less than 5 spinal anesthesia experience and at the lower end of the learning spectrum.

Third, the potential learning effect over the course of the study could be present; however, it was mitigated by performing all spinal blocks in a row and minimizing the number of procedures done outside study. Yet, it must be noted that as per Kopacz et al., notable improvement in the spinal anesthesia technique among novice residents require at least 20 procedures to be performed [17]. In our study, 12 residents performed each 12 spinal blocks and only 2 residents performed each 18 spinal blocks, all of which are below minimal required number.

Finally, we found some challenges with the use of the US-assisted approach in general: the difficulty to mark the point for needle insertion that can change if the patient moves between the time of skin marking and the actual procedure, the tissue distortion particularly in the elderly with mobile and loose skin, and to guarantee that the trainee will follow the exact direction of the probe despite the clear instruction about the angle at which the needle should be inserted. It is important to highlight on the different definitions of attempts, passes, and redirections in the literature as a redirection without withdrawal is sometimes considered in some reports as an additional attempt, thus making comparison of measured outcomes difficult among studies.

## Conclusions

In conclusion, our study showed that preprocedural US scanning did not improve the ease of midline and paramedian spinal anesthesia as compared to the conventional landmark midline technique when performed by junior residents in an elderly population. Thus, there was no observed additional value to preprocedural US, whether using the TM or the PSO views for anesthesiologists in training. Our results should be confirmed with the use of the newer modality of real-time US guidance in the future.

## Data Availability

The datasets used and/or analyzed during the current study are available from the corresponding author on reasonable request.

## Abbreviations

CA-1: First year clinical anesthesia residents
LFD: Ligamentum flavum-dura meter complex
LM: Landmark-guided midline
PLL: Posterior longitudinal ligament
PSO: Parasagittal oblique
TM: Transverse median
UM: Ultrasound guided midline technique
UP: Ultrasound guided paramedian
US: Ultrasound
